# Chronic kidney disease in Sri Lanka: Health systems challenges of patients on hemodialysis

**DOI:** 10.1002/puh2.155

**Published:** 2024-01-24

**Authors:** D. C. R. Weerakoon, E. P. E. D. Z. Siriwardana, J. M. K. B. Jayasekara, H. D. W. T. Damayanthi, Thinley Dorji, Don Eliseo Lucero‐Prisno

**Affiliations:** ^1^ Department of Medical Laboratory Sciences Faculty of Allied Health Sciences General Sir John Kotelawala Defence University Werahera Sri Lanka; ^2^ Department of Nursing Faculty of Allied Health Sciences University of Peradeniya Peradeniya Sri Lanka; ^3^ Department of Internal Medicine Central Regional Referral Hospital Gelephu Bhutan; ^4^ Department of Global Health and Development London School of Hygiene and Tropical Medicine London UK

**Keywords:** kidney diseases, population health, quality of life, renal replacement therapy, Sri Lanka

## Abstract

Chronic kidney disease has now attained epidemic proportions, placing a significant strain on Sri Lanka's healthcare system. The North Central Province of Sri Lanka has been afflicted by this disease for decades, confronting multiple challenges. Hemodialysis is the primary form of renal replacement therapy available to end‐stage renal disease patients in Sri Lanka. Providing hemodialysis sessions free of cost in the government health sector comes with major costs on the healthcare system. As a country with a low‐to‐middle income status undergoing through reeling economic crisis, providing free healthcare in government hospitals and increasing the capacity and quality of treatment facilities for the growing number of patients with chronic kidney disease will remain a major challenge for the coming decade. The high cost for a single dialysis session, lack of resources and workforce to meet demand, occupational barriers of patients, and the economic burdens including out‐of‐pocket expenditures significant barriers in achieving quality treatment sessions and the quality of life of patients. In addition, the absence of a consistent screening program has contributed to the progression of the disease ending up requiring renal replacement therapy. This article suggests potential strategies that can be implemented to mitigate the aforementioned problems and enhance the overall health, quality of life, and survival of patients with chronic kidney disease and those who receive hemodialysis.

## INTRODUCTION

In 2019, the Annual Health Statistics Report listed urinary system illnesses, including chronic kidney disease (CKD), as the fourth leading cause of hospitalization and also the sixth leading cause of hospital mortality in Sri Lanka [1]. According to data from 2020, a total of 164,000 individuals were diagnosed with CKD. Furthermore, a comprehensive study indicates that approximately 10% of the population in Sri Lanka is afflicted by kidney‐related ailments. There has been a notable increase in the burden of CKD in the country in the last two decades with the identification of Chronic Kidney condition of Unknown etiology (CKDu) cases among a population residing in the North Central Province [2], specifically in the Anuradhapura and Polonnaruwa districts. The occurrence of CKD in these regions varies between 5% and 15% [3]. According to statistical data from 2020, the gravity of this problem is underscored by a notable increase in the annual mortality rate, with around 10,500 deaths attributed to renal failure and diseases of the kidneys [4]. This indicates that a considerable portion of the population in Sri Lanka lacks the ability to effectively manage the diagnosis of CKD and other kidney diseases. This deficiency in correct management contributes to a significant number of fatalities within the country.

The disease primarily impacts those in the agricultural sector who are in the younger and middle age categories [2]. CKD can progress to end‐stage renal disease (ESRD), where the patients need to rely on renal replacement therapy (RRT) such as renal transplantation, hemodialysis, or peritoneal dialysis. Hemodialysis serves as the primary modality for RRT among patients with ESRD in Sri Lanka [5] and is predominantly accessible within tertiary care facilities situated in larger urban areas. In the year 2020, Sri Lanka documented a significant figure of 9500 individuals who received dialysis therapy. This data serves as a clear indication that a substantial portion of the population experiences CKD and requires dialysis as a temporary intervention to mitigate mortality risks [4].

Sri Lanka possesses a comprehensive healthcare system that provides unrestricted access to healthcare services. The majority of dialysis units that get state funding are located within hospitals, whereas only a limited number of independent dialysis units exist. The majority of these healthcare facilities are facing significant challenges due to the high volume of patients in need of chronic RRT.

## CHALLENGES FOR HEMODIALYSIS IN THE NORTH CENTRAL PROVINCE

### Lack of resources and workforce

The majority of the healthcare facilities are facing a significant burden due to the large influx of patients. According to the latest records from the Ministry of Health, there are only 8 hemodialysis centers currently functioning in Anuradhapura and Polonnaruwa districts, with less than 100 dialysis machines for the treatment of over 1000 patients (Table 1) [6]. As a consequence, many of these units are compelled to prioritize dialysis treatment for individuals who have kidney transplant plans and younger patients with fewer concurrent medical conditions [5]. Unfortunately, this prioritization strategy has led to an increased number of reported fatalities.

**TABLE 1:** Distribution of service utilization of hemodialysis facilities by district in government hospitals of Sri Lanka, 2020 [6].

Province	District	No. of government hospitals with HD units	No. of HD units	No. of HD machines	HD machines per 100,000 population
	Kandy	3	5	91	6.6
	Matale	1	1	8	1.7
**Central**	Nuwara Eliya	1	1	2	0
Sub total		5	7	101	
	Ampara	2	2	18	4.3
	Batticaloa	4	4	10	2.47
**Eastern**	Kalmunai	4	4	13	4.48
	Trincomalee	4	4	17	
Sub total		14	14	58	
	Jaffna	4	4	20	3.4
	Kilinochchi	1	1	2	1.76
**Nothern**	Mannar	1	1	2	2
	Mullaitivu	1	1	3	3.25
	Vavuniya	2	2	12	6.97
Sub total		9	9	39	
**North central**	Anuradhapura	5	5	49	5.69
	Polonnaruwa	3	3	33	8.13
Sub total		8	8	82	
**North Western**	Kurunegala	6	6	34	2.1
	Puttalam	1	1	5	0.7
Sub total		7	7	39	
	Galle	2	2	13	1.2
**Southern**	Hambantota	3	3	6	1
	Matara	2	2	7	0.9
Sub total		7	7	26	
**Uva**	Badulla	3	3	22	2.7
	Monaragala	2	2	12	2.66
Sub total					
		5	5	34	
	Colombo	7	8	67	2.88
**Western**	Gampaha	4	4	20	0.87
	Kalutara	3	3	11	0.9
Sub total		14	15	98	
	Kegalle	0	0	0	
**Sabaragamuwa**	Rathnapura	3	3	15	1.38
Western	Sub total	3	3	15	
Total		72	75	492	2.5

A significant proportion of patients receive dialysis only twice per week or less, although they require a frequent dialysis of three times per week. The excessive expenses borne by the government for each individual dialysis session, along with the overcrowding of patients and scarce resources, have severely hindered hospitals from meeting this demand. This has had a significant impact on the quality of life and prognosis of patients, resulting in severe complications and death. Furthermore, individuals requiring hemodialysis therapy for their medical condition experience delays in initiating dialysis as a result of above limitations, hence posing a significant risk to their health and prognosis.

According to the study by Ranasinghe et al., the mean cost of a dialysis session per person in Sri Lanka was LKR 6377 (US$19.55) [5]. The yearly expense for hemodialysis treatment, which involves two to three 4‐h sessions per week, for a patient with chronic renal failure, ranged from LKR 663,208–994,812 (equivalent to US$2032.81–3049.21). Additionally, it was shown that expenses related to drugs and consumables were 70.4%–84.9% of the overall costs, with the salary of the nursing staff at each unit accounting for 7.8%–19.7% [5].

In addition to the free treatments offered by governmental hospitals, a limited number of privately held institutions also provide hemodialysis services. But these resources are primarily concentrated in the Western province of the country and are scarcely present in rural areas such as the North Central Province [7]. However, patients are required to pay approximately LKR 8000 (US$24.41) for each individual dialysis session. If these therapies are administered twice weekly, the total monthly expense would reach LKR 64,000 (US$195.27), approximately to the average monthly household income in the North Central Province of Sri Lanka, based on 2019 statistics from the Central Bank of Sri Lanka (2023) [8]. Therefore, the availability of these services is limited to patients who possess the financial capacity to personally cover the related expenses, which represents an extremely insignificant fraction in comparison to the overall ESRD population in the nation.

Inadequately trained personnel are a significant barrier to the expansion and growth of dialysis services in the nation. There are only 35 nephrologists in the country, a ratio of 1.6 per million people [9]. There are no dialysis technicians or designated personnel involved in the provision of dialysis care, which is provided solely by nurses. At present, there is no established official training program for dialysis nurses. The majority of training opportunities are provided through seminars and educational sessions organized by particular institutions and the Sri Lanka Society of Nephrology [7].

Arteriovenous fistulas (AVFs) are typically performed by vascular surgeons, accessible predominantly in select larger institutions located in major metropolitan areas. Due to a high volume of cases and a dearth of surgical theatres, there are significant delays in the creation of AVFs. Studies indicate that the majority of patients begin dialysis treatment with temporary vascular catheters, which is regarded as a suboptimal method due to the associated inconvenience and the substantial risk of central‐line‐associated bloodstream infections [10, 11].

In addition, the scarcity of equipment and facilities for kidney transplantation is an additional challenge for kidney patients in Sri Lanka. Currently, there are only 10 government hospitals on the entire island that have this facility for kidney transplantation, indicating that it is difficult to even schedule a transplant because most patients must wait for an extended period of time. Without the expense of long‐term medication, the total cost of a transplant is approximately LKR 2500,000 (US$7719.27) [4].

### Economic status and occupations of patients

Economic constraints are another hurdle that stands in the way of these patients toward better health. North Central Province is the main location of paddy farming in the country, and the majority of the population is farming communities. According to the statistics of 2019, the average monthly income per individual in the North Central Province is LKR 18,131 (US$55.57) and that of per household is LKR 64,645 (US$198.14) [8]. In fact, their income is inherently unpredictable both within and across years, as it is contingent upon the fluctuating paddy yield each season, which is influenced by weather conditions and the economic state of the country. Consequently, the challenges associated with CKD and the expenses of therapies have exacerbated the hardships they face.

Owing to the excessive expenses associated with private dialysis centers, many are hesitant to use these services. Moreover, given that almost all of these facilities are mostly situated in major commercial areas, individuals are required to incur additional costs for transportation and lodging. Consequently, the majority of patients must restrict their dialysis sessions to a minimum and some may have to postpone the start of their treatments due to overcrowded government institutions. Despite receiving dialysis treatments from a government facility, many individuals encounter challenges in covering expenses for other medications, laboratory tests, transportation fees, and associated costs.

It has been found that in many cases, individuals with renal disease often serve as the primary earners for their families. Therefore, in cases where the primary breadwinner of a household develops kidney disease, the overall financial stability of the family is compromised. The government is currently failing to deliver essential pharmaceuticals required for dialysis treatment and transplantation. This suggests that individuals with renal illness will need to get these treatments through private means, which might be financially burdensome.

Compared to other regions of the country, CKDu in the North Central Province mostly affects younger and middle‐aged farmers [2], whereas agriculture is the only source of income for most people. Most families in these regions have farmed for generations and face major socioeconomic challenges in switching careers. Many people also lack the skills and education to switch to alternative career options. Males in most families generate income, whereas females care for children and domestic duties. The early engagement of males in this profession might be associated with occupational exposure and a higher propensity for the early onset of kidney impairment.

### Transport distance and cost for the patients

The hemodialysis centers in Anuradhapura and Polonnaruwa are located at tens of hundreds of kilometers for rural residents. This long travel is physically and financially challenging. Given the prevalence of poor financial standing in these communities, people are less inclined to devote significant time and money to medical treatments. Therefore, despite following dialysis treatment recommendations, patients sometimes attend only one session to save money, resulting complications and early mortality.

### Unavailability of a proper screening and management program

Given the high costs required for dialysis therapy, it is essential to reduce the number of patients who progress to ESRD. This can be accomplished through the implementation of a systematic and consistent screening program intended at early identification and facilitating effective management strategies. The screening program currently being carried out by the Ministry of Health in the high‐risk populations of the country has not achieved the expected results, especially for the population of the North Central Province. Furthermore, there is no precise patient register in place to facilitate patient monitoring and administration. This is primarily due to deficiencies in organization, technical capabilities, resource allocation, and a lack of consistency and improvement. Therefore, there is a need for a screening and management program using reliable and expeditious methods for lowering the rates of morbidity and mortality among those affected by CKD/CKDu [12].

### Poor knowledge and awareness of the community

Management and care of dialysis patients includes appropriate diets, monitoring of hydration, medication regimens, and frequent medical follow‐up. Many patients disregard medical advice and neglect scheduled treatment sessions, citing a variety of beliefs. Family members are also unaware and do not help in patient follow‐up [13]. This is an important sector requiring timely efforts in improving health literacy on CKD.

## RECOMMENDATIONS FOR FUTURE DIRECTIONS

To improve the well‐being and overall survival of patients with CKD and those undergoing dialysis treatment, there is a need to increase the number of dialysis centers and machines as well as optimize the dialysis sessions of patients including their management procedure. There should be timely referrals, predialysis counseling, and timely access to AVF surgeries. The expansion of the dialysis workforce is necessary in this context including the introduction of “dialysis assistants/technicians.” Given the numerous difficulties linked to hemodialysis, it would be advantageous to assess the effectiveness and feasibility of establishing and improving a Peritoneal Dialysis (PD) program.

Peritoneal dialysis is significantly underutilized in Sri Lanka as a method of long‐term RRT. The majority of patients are hesitant to embrace peritoneal dialysis because they are afraid to assume the duty of independently administering the treatment. Furthermore, the underutilization of peritoneal dialysis might be attributed to a shortage of skilled personnel, lack of knowledge, and awareness among the people, disruptions in the availability of dialysis solutions and catheters, rising expenses, and inadequate reimbursement for [7]. Nevertheless, it is now crucial to prioritize the implementation of a highly efficient PD program to reduce the strain on dialysis clinics and improve the overall health and prognosis of patients who endure lengthy waiting lists for treatment.

It is crucial that the healthcare system prioritizes the prevention and early detection of CKD especially populations at high risk, such as those with diabetes and hypertension, as well as those living in regions where CKDu is prevalent. The primary aim of this approach is to delay the progress to ESRD.

Hence, it is crucial to establish a more efficient and methodical screening strategy, accompanied by subsequent surveillance and containment measures, specifically focusing on regions with a high disease frequency. The current screening program employs a range of laboratory procedures to identify individuals at risk, such as monitoring blood pressure, assessing urine albumin creatinine ratio using an early morning urine sample, and testing serum creatinine levels to estimate glomerular filtration rate [14]. Therefore, they may employ mobile laboratory services to improve the effectiveness of the project. Furthermore, it is imperative to improve the management of individuals who have been identified through postscreening and continuous monitoring, with the aim of minimizing the progression of the disease in patients. This can be achieved by regular laboratory examinations and the establishment of an effective management plan, beginning with proper documentation and an accurate patient register. Furthermore, it is crucial to enhance the quality and the sensitivity of the screening tools by promoting evidence‐based research.

**FIGURE 1:**
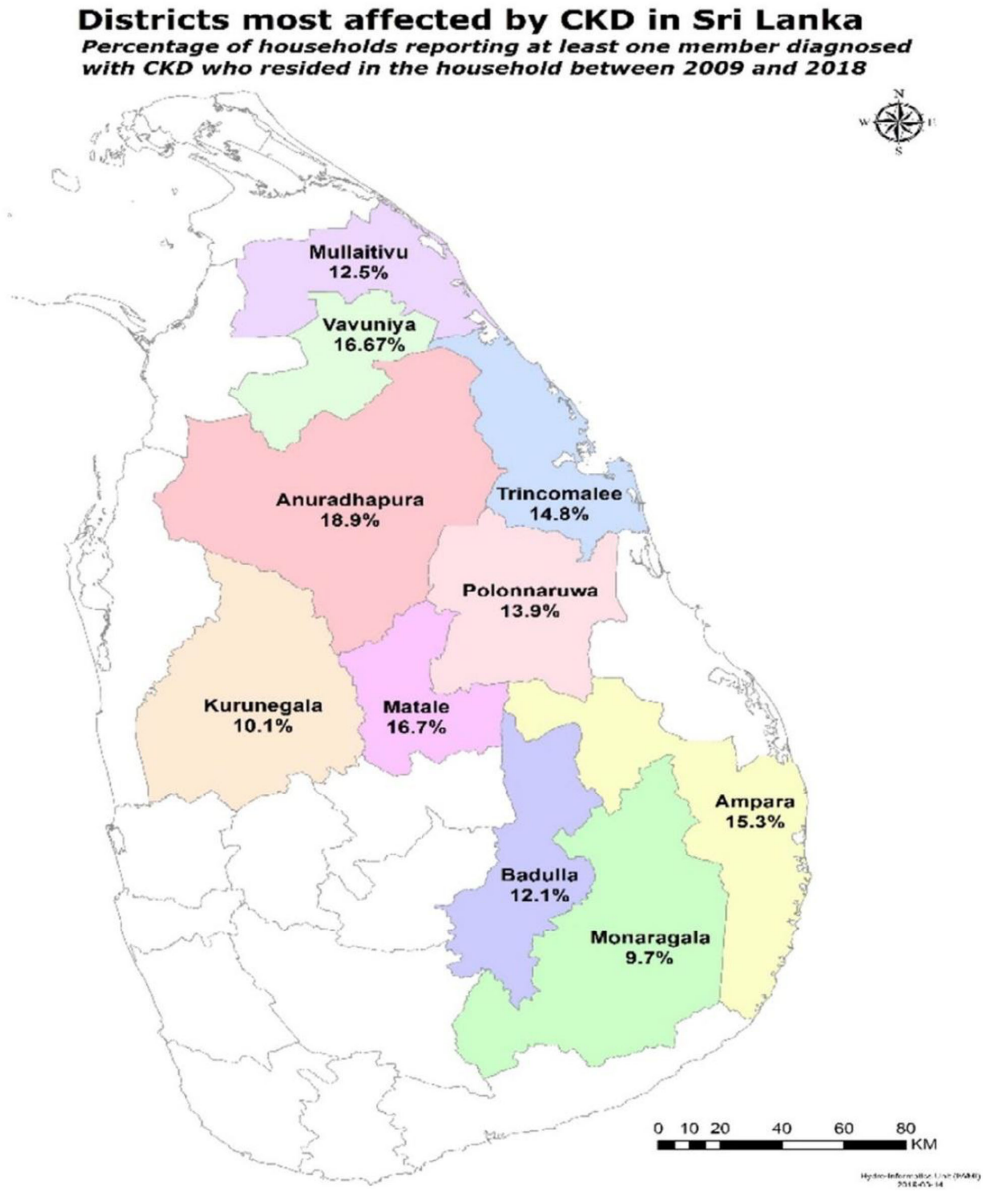
Chronic kidney disease (CKD) prevalence rates across the most affected districts in Sri Lanka, 2009–2016 [9].

**FIGURE 2:**
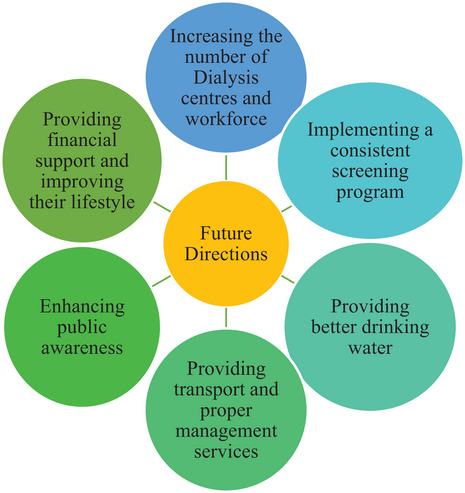
Recommendations for improving the health and quality of life of patients with chronic kidney disease and receive hemodialysis in Sri Lanka.

Moreover, following the initial identification of the disease in the North Central Province, there has been a consistent increase in its prevalence in the neighboring provinces (Figures 1 and 2). Therefore, it is crucial to determine the causes and main factors responsible for the disease in these regions, in order to develop and execute effective prevention measures and strategies to reduce the course of the disease.

Research studies and investigations have demonstrated that water plays a significant role in the incidence of kidney disorders, particularly in the North Central Province [12]. In recent years, the government has implemented reverse osmosis (RO) facilities in a number of CKDu‐affected communities. This initiative has produced notable and beneficial results. Consequently, it is essential to expand the scope of these initiatives to include additional communities located in all affected districts.

The amount of government funds allocated to patients for medication and other costs associated with kidney disorders is insufficient. Therefore, it would be optimal for the dialysis center to provide transportation services to and from patients’ homes. This will help ensure compliance to hemodialysis sessions.

The dialysis unit should have designated spaces where patients can relax and be monitored after dialysis treatments to help them recover until their normal physical and mental states are restored. Sri Lanka's healthcare system places much less emphasis on posttreatment care services, particularly in the context of dialysis treatments. Nutrition advice, water restriction, and diet compliance are also important. Patients’ hemodialysis treatments and care must be tailored to their age, medical conditions, comorbidities, and lifestyle.

Improper management of health problems sometimes leads to a significant percentage of people undergoing discomfort and complications. Patients and the community are advised to consistently use purified and filtered water from RO facilities. Moreover, it is essential for individuals to consistently maintain proper hydration, especially when engaging in strenuous tasks like farming, in order to avoid dehydration. These procedures are essential for the prevention of the disease and the management of its advancement. Increasing patients’ and carers’ understanding of the condition is crucial for effectively managing CKD in the country. Individuals should be informed about the significance of being mindful of their normal dietary habits, fluid intake, reducing strenuous physical activity, and minimizing exposure to extreme temperatures, among other measures, in order to avoid and control the advancement of the condition. Therefore, it is crucial to develop programs that provide education to patients and villagers regarding the many risk factors, potential complications, preventive measures, and suitable therapy options related to CKD and hemodialysis.

## CONCLUSION

Sri Lanka is burdened by CKD and its consequences for decades, and the costs and other issues associated with hemodialysis services provided to ESRD patients have exacerbated this burden. In light of the country's economic situation, the greatest challenge for the healthcare system has been the provision of adequate, high‐priced hemodialysis services to an ever‐increasing population of patients. In addition, the dearth of adequate resources and workforce, the economic status and occupation of patients, the transport distance and costs for patients, the absence of a proper screening program, and the lack of community awareness and knowledge have negatively impacted the service and the prognosis of patients. Therefore, it is crucial to pursue and implement strategies to enhance the quality standards of this life‐saving procedure in order to improve the patients’ quality of life and overall health.

## AUTHOR CONTRIBUTIONS

*Conceptualization; investigation; writing—original draft; methodology; writing—review and editing; validation; visualization; formal analysis; resources*: D. C. R. Weerakoon. *Conceptualization; investigation; validation; visualization*: E. P. E. D. Z. Siriwardana. *Conceptualization; validation; writing—review and editing; formal analysis; supervision; methodology; resources; project administration*: J. M. K. B. Jayasekara. *Conceptualization; writing—review and editing; methodology; validation; formal analysis; resources; supervision; project administration*: H. D. W. T. Damayanthi. *Writing—review and editing; validation; formal analysis; supervision; project administration*: Thinley Dorji. *Writing—review and editing; validation; formal analysis; project administration; supervision*: Don Eliseo Lucero‐Prisno.

## CONFLICT OF INTEREST STATEMENT

TD and DELP are members of the editorial board of this journal. They have been blinded and excluded from all processes related to the peer review of this journal.

## Data Availability

Data sharing not applicable to this article as no datasets were generated or analyzed during the current study.

## References

[1] Ministry of Health . Sri Lanka Medical Statistics Unit Ministry of Health. Ministry of Health. 2019.

[2] Jayasekara KB , Dissanayake DM , Sivakanesan R , et al. Epidemiology of chronic kidney disease, with special emphasis on chronic kidney disease of uncertain etiology, in the north central region of Sri Lanka. J Epidemiol. 2015;25(4):275‐280. doi:10.2188/jea.je20140074 25787679 PMC4375281

[3] Wijewickrama ES , Thakshila WAG , Ekanayake EMD , et al. Prevalence of CKD of unknown etiology and its potential risk factors in a rural population in Sri Lanka. Kidney Int Rep. 2022;7(10):2303‐2307. doi:10.1016/j.ekir.2022.07.012 36217515 PMC9546732

[4] Heiyantuduwa S . Chronic Kidney Disease and Sri Lanka: Introduction. SLK Foundation; 2022. https://slkfoundation.co.uk/02/15/2023/provide‐healthy‐food‐2/

[5] Ranasinghe P , Perera YS , Makarim MF , Wijesinghe A , Wanigasuriya K . The costs in provision of haemodialysis in a developing country: a multi‐centered study. BMC Nephrology. 2011;12(1):42. doi:10.1186/1471-2369-12-42 21896190 PMC3189097

[6] Ministry of Health . 2020 Annual Health Bulletin Ministry of Health Sri Lanka . Ministry of Health; 2020.

[7] Wijewickrama ES , Herath N . Global dialysis perspective: Sri Lanka. Kidney360. 2022;03:1603‐1606. doi:10.34067/kid.0001592022 PMC9528390 36245652

[8] Central Bank of Sri Lanka . Sri Lanka Socio‐Economic Data 2023 . Central Bank of Sri Lanka; 2023.

[9] Kafle K , Balasubramanya S , Horbulyk T . Prevalence of chronic kidney disease in Sri Lanka: a profile of affected districts reliant on groundwater. Sci Total Environ. 2019;694(133767):133767. doi:10.1016/j.scitotenv.2019.133767 31756806

[10] Weldetensae MK , Weledegebriel M , Tesfahunegn A , Berhe E , Gebrearegay H . Catheter‐related blood stream infections and associated factors among hemodialysis patients in a tertiary care hospital. Infect Drug Resist. 2023;16(16):3145‐3156. doi:10.2147/idr.s409400 37249964 PMC10216862

[11] Nasiri E , Rafiei MH , Mortazavi Y , Tayebi P , Ghasemzadeh Bariki M . Causes and risk factors of hemodialysis catheter infection in dialysis patients: a prospective study. Nephro‐Urol Mon. 2022;14:e117820. doi:10.5812/numonthly.117820

[12] Rajapakse S , Shivanthan MC , Selvarajah M . Chronic kidney disease of unknown etiology in Sri Lanka. Int J Occup Environ Health. 2016;22(3):259‐264. doi:10.1080/10773525.2016.1203097 27399161 PMC5102238

[13] Seneviratne B , Wijayasiri W , Jayaweera S , Kumara K . Knowledge, attitudes & practices on chronic kidney disease among people of the north central province of Sri Lanka. Br J Med Health Res. 2016;3(10):108‐121. https://www.bjmhr.com/download.php?filename=bjmhr_admin/uploads/publish_pdf/155__1477711363310010.pdf&split=32&art_map_id=155

[14] Epidemiology Unit Ministry of Health . Screening Guidelines Chronic Kidney Disease Sri Lanka 2017. Epidemiology Unit Ministry of Health. 2017.

